# Genomic Characterization of Tilapia Lake Virus Isolates Recovered from Moribund Nile Tilapia (*Oreochromis niloticus*) on a Farm in the United States

**DOI:** 10.1128/MRA.01368-19

**Published:** 2020-01-23

**Authors:** Mohammad Shamim Ahasan, William Keleher, Cem Giray, Brenda Perry, Win Surachetpong, Pamela Nicholson, Lowia Al-Hussinee, Kuttichantran Subramaniam, Thomas B. Waltzek

**Affiliations:** a Department of Infectious Diseases and Immunology, College of Veterinary Medicine, University of Florida, Gainesville, Florida, USA; b Emerging Pathogens Institute, University of Florida, Gainesville, Florida, USA; c Faculty of Veterinary and Animal Sciences, Hajee Mohammad Danesh Science and Technology University, Dinajpur, Rangpur, Bangladesh; d Kennebec River Biosciences, Richmond, Maine, USA; e Center for Advanced Studies for Agriculture and Food, Kasetsart University Institute for Advanced Studies, Kasetsart University, Bangkok, Thailand; f Department of Veterinary Microbiology and Immunology, Faculty of Veterinary Medicine, Kasetsart University, Bangkok, Thailand; g Next Generation Sequencing Platform, University of Bern, Bern, Switzerland; KU Leuven

## Abstract

Here, we present the complete coding sequences of two tilapia lake virus (TiLV) isolates recovered during an investigation of a mortality event in farmed Nile tilapia in the United States. Phylogenetic analysis supported the isolates as each other’s closest relatives and members of a clade of Thai TiLV strains.

## ANNOUNCEMENT

**FIG 1:**
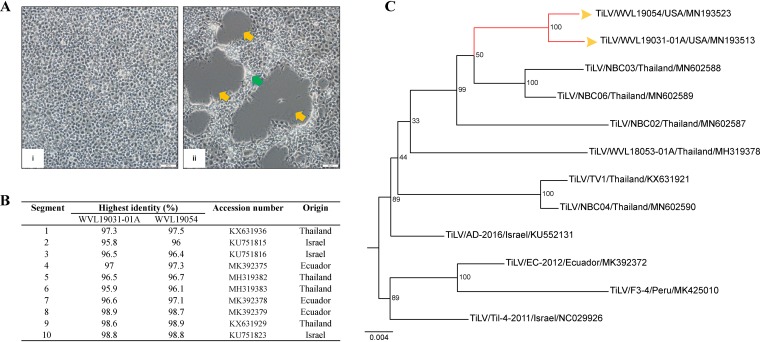
(A) Striped snakehead (SSN-1; E-11 subclone) cells inoculated with internal tissue homogenates. (i) Uninfected control. (ii) Infected SSN-1 cultures displaying multiple plaques (yellow arrows) and associated vacuolated cells (green arrow) at the edge of the plaques. Bar, 50 μm. (B) The table represents the highest nucleotide identity for each gene segment of the U.S. TiLV isolates (WVL19031-01A and WVL19054) to TiLV strains present in the GenBank database. (C) Maximum likelihood phylogram depicting the relationship of the U.S. TiLV isolates (yellow arrowheads) to 10 other TiLV isolates based on the nucleotide sequences of the PB1 gene. Bootstrap values are given at each node, and the branch lengths represent the number of inferred substitutions as indicated by the scale.

Tilapia lake virus (TiLV), member of the recently established genus Tilapinevirus and family Amnoonviridae (1), is a globally emerging virus affecting cultured Nile tilapia and hybrids (2). The TiLV genome has 10 segments of negative-sense single-stranded RNA (1). Here, we present the complete coding sequences of two TiLV isolates (WVL19031-01A and WVL19054) recovered during an epidemiological investigation of a suspected TiLV outbreak in a U.S. tilapia facility in Idaho in February 2019. The isolates were recovered from separate tissue homogenates of pooled internal organs (kidney/spleen/liver) from 5 fish that were inoculated onto confluent monolayers of the striped snakehead cell line (SSN-1; E-11 subclone). Cytopathic effects were observed 4 to 10 days postinoculation (Fig. 1A). Viral genomic RNA was extracted from each SSN-1 cell culture supernatant using an RNeasy minikit (Qiagen). The cDNA libraries were generated using the NEBNext Ultra RNA library prep kit (New England Biolabs) and sequenced on an Illumina MiSeq sequencer using a MiSeq reagent kit v3 (600-cycle) (3). In total, 8,055,592 and 1,954,170 high-quality reads (average read length, 214 bp) were obtained for WVL19031-01A and WVL19054, respectively, after removing low-quality reads and quality trimming in CLC Genomics Workbench v10.1.1 using default parameters. *De novo* assembly of the paired-end reads was performed in SPAdes v3.10.0 with default parameters (4). BLASTN analysis of the assembled contigs against the National Center for Biotechnology Information (NCBI) nonredundant protein database recovered all 10 segments for both isolates with the highest identities to each other (>99%; data not shown) and lower identities to previously sequenced strains of TiLV (95.8 to 98.9%; Fig. 1B). The total length of the complete coding sequence for both WVL19031-01A and WVL19054 was 9,051 bp. The G+C contents of WVL19031-01A and WVL19054 were 47.7% and 47.8%, respectively, with average coverages of 3,376 and 445 reads/nucleotide, respectively. The integrity of the TiLV sequences were verified by mapping the reads in Bowtie 2 (5) and visually inspecting the alignments in Tablet 1.17.08.17 (6). Putative open reading frames for the TiLV genomes were predicted using GeneMarkS (http://exon.gatech.edu/GeneMark/genemarks.cgi) (7), restricting the search to virus sequences. The 5′ and 3′ untranslated regions of the 10 segments of TiLV WVL19031-01A and WVL19054 were not determined. Maximum likelihood phylogenetic analysis performed in MEGA X (8), based on the nucleotide alignment of the TiLV PB1 gene sequences, supported TiLV WVL19031-01A and WVL19054 as each other’s closest relatives and members of a previously determined clade of Thai TiLV strains (3, 9) (Fig. 1C).

This study represents the first detection of TiLV in farmed Nile tilapia in North America. The farm had a history of importing live tilapia from Thailand, and our phylogenetic analysis of the U.S. TiLV isolates supported them being most closely related to Thai TiLV strains. The detection of TiLV in farmed Nile tilapia in the United States underscores the immediate need for a surveillance program coordinated by national/state authorities to curb the threat that TiLV poses to the U.S. tilapia aquaculture industry.

### Data availability.

Complete coding sequences and raw sequence data of TiLV WVL19031-01A and WVL19054 have been deposited in NCBI GenBank under accession no. MN193513 through MN193522 and MN193523 through MN193532 and the Sequence Read Archive (SRA) under accession no. SRX6878421 and SRX6878422, respectively.
